# Correspondence: Revisiting the theoretical cell membrane thermal capacitance response

**DOI:** 10.1038/s41467-017-00435-5

**Published:** 2017-11-10

**Authors:** Michael Plaksin, Eitan Kimmel, Shy Shoham

**Affiliations:** Faculty of Biomedical Engineering & Russell Berrie Nanotechnology Institute, Technion – Israel Institute of Technology, Haifa, 32000 Israel

## Introduction

**Fig. 1:**
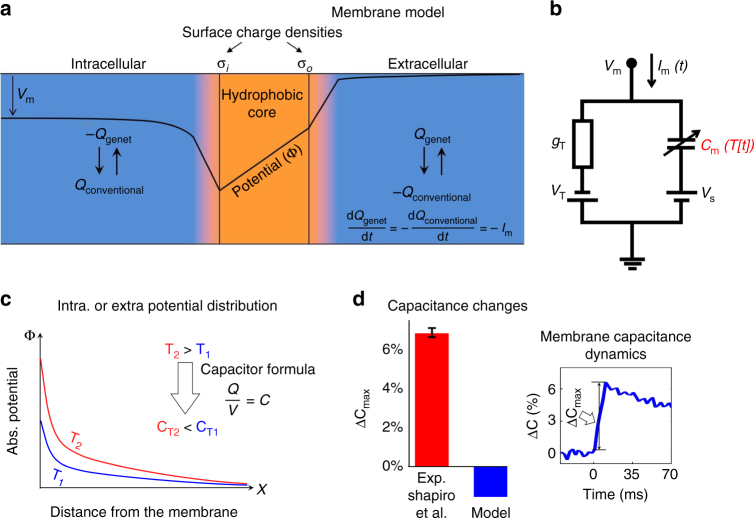
Predicted membrane electrical capacitance after temperature increase. **a** Theoretical Gouy–Chapman–Stern (GCS) model which includes phospholipid (hydrophobic) core and extra-membranal boundary sub-regions. The model is shown under conventional charge notation^9^ and the non-standard notation of Genet et al.^1, 6^, where the charge derivative has opposite direction to the current convention^9^. **b** Membrane-equivalent electrical circuit reproduced from Shapiro et al.^1^; the membrane current is marked according to the convention^1, 9^. **c** Illustration of potential distribution at the extra-membranal boundary regions. Temperature elevation leads to higher potential gradients, which according to the classical capacitor formula corresponds to a reduction in capacitance of the extra-membranal regions. **d** Discrepancy between capacitance measurement (reproduced from Shapiro et al.^1^) and sign-corrected model simulations (PE:PC bilayers^1^, laser parameters: duration −10 ms, energy −7.3 mJ)

Shapiro et al.^1^ have shown that the thermal transients generated when short wave infrared light is absorbed in neural tissue are accompanied by an increase of the cell membrane’s electrical capacitance. This capacitance change elicits capacitive membrane currents which are unrelated to any specific ion channels and which can explain infrared neural stimulation (INS) and a variety of other thermal neurotechnologies. Their findings on the thermal capacitance increase have since been supported by experiments from several additional groups^2–5^, thus becoming an important contribution to our understanding of thermal neurostimulation. In addition to their primarily experimental study, Shapiro et al.^1^ also suggested a theoretical explanation of the increase in capacitance with temperature, in which the membrane capacitance was modeled according to the principles of Gouy–Chapman–Stern theory^1, 6^. In this theoretical approach, the membrane capacitance is determined by calculating the overall capacitance of the phospholipid core region in series with the capacitances of the intracellular and extracellular extra-membranal boundary regions; ionic concentrations are obtained when diffusion and electrical forces on the different ions reach equilibrium (following a Poisson–Boltzmann distribution, Fig. 1a).

As implemented by Shapiro et al.^1^, this model demonstrated agreement with experimentally measured currents. However, this seemingly complete theoretical explanation, and several subsequent analyses by other groups^7, 8^ who closely followed it, are incorrect due to a modeling error. Intuitive energetic considerations regarding the ionic double layer capacitance on each side of the membrane predict that in order to maintain equilibrium, the additional thermal energy input will be offset by correspondingly higher electrical energy and absolute potentials (Fig. 1c). This stronger potential difference corresponds to a capacitance decrease, which is opposite to the experimental measurements. We have traced the modeling error back to a reliance on Genet et al.^6^ non-standard notation for membrane mobile charge (see also Fig. 1a): “Let *Q* denote the total capacitive charge in the extracellular space (the total charge within the cytoplasm will be –*Q*; *Q* > 0 for a resting cell)”^6^. Under this notation (also used by several related theoretical studies^7, 8^), the derivative of the mobile membrane charge is $\frac{{{\mathrm{d}}Q}}{{{\mathrm{d}}t}} = {I_{{\mathrm{Genet}}}} = - {I_{\mathrm{m}}}$, where *I*_m_ is a membrane current conventionally defined positive for a current flowing from the intracellular to the extracellular domain^9^, charging the intracellular side of the membrane capacitor. Our reanalysis using a sign-corrected model predicts a decrease, rather than an increase in membrane capacitance with temperature (Fig. 1d).

Given this discrepancy, a revised explanation for the membrane capacitance’s thermal increase observed in Shapiro et al.’s key experiments^1^ is therefore needed. By examining the biophysics literature on thermal membrane effects^10–12^, we identified membrane structural dimensional changes as a plausible, predictive mechanism for this phenomenon. This topic and its theoretical and predictive (validation) consequences are explored in Plaskin et al.^13^.
